# CCL2/CCR2 axis is associated with postoperative survival and recurrence of patients with non-metastatic clear-cell renal cell carcinoma

**DOI:** 10.18632/oncotarget.10492

**Published:** 2016-07-08

**Authors:** Zewei Wang, Huyang Xie, Lin Zhou, Zheng Liu, Hangcheng Fu, Yu Zhu, Le Xu, Jiejie Xu

**Affiliations:** ^1^ Department of Biochemistry and Molecular Biology, School of Basic Medical Sciences, Fudan University, Shanghai, China; ^2^ Department of Urology, Fudan University Shanghai Cancer Center, Shanghai, China; ^3^ Department of Oncology, Shanghai Medical College, Fudan University, Shanghai, China; ^4^ Department of Urology, Zhongshan Hospital, Fudan University, Shanghai, China; ^5^ Department of Urology, Ruijin Hospital, Shanghai Jiao Tong University School of Medicine, Shanghai, China

**Keywords:** clear-cell renal cell carcinoma, CCL2, CCR2, prognostic biomarker, overall survival

## Abstract

**Purpose:**

Chemokine (C-Cmotif) ligand 2 (CCL2) is a major chemokine that recruit monocytes and macrophages to the sites of inflammation. Recent researches have clarified that overexpression of CCL2 is associated with unfavorable prognosis in various cancer types. In this study, we aim to determine the prognostic value of CCL2 expression as well as its receptor C-C motif receptor type 2 (CCR2) in patients with non-metastatic clear cell renal cell carcinoma (ccRCC) after surgery.

**Results:**

Both high CCL2 and CCR2 expression were remarkably correlated with shortened survival time (*P* < 0.001 and *P* < 0.001, respectively) and increased risk of recurrence (*P* = 0.001 and *P* = 0.003, respectively). The combination of CCL2 and CCR2 expression (CCL2/CCR2 signature) could offer a better prognostic stratification. Furthermore, multivariate analyses identified CCL2/CCR2 signature as an independent risk factor for overall survival (OS) and recurrence-free survival (RFS) (*P* = 0.007 and *P* = 0.043, respectively). The incorporation of CCL2/CCR2 signature would refine individual risk stratification and predictive accuracy of the well-established models.

**Materials and Methods:**

We retrospectively examined the intratumoral expression of CCL2 and CCR2 by immunohistochemical staining in 268 histologically proven non-metastatic ccRCC patients receiving surgery in a single institution between 2001 and 2004. Kaplan-Meier analysis and Cox regression were applied to determine the prognostic value of CCL2 and CCR2 expression. Concordance index was calculated to compare predictive accuracy of the established models.

**Conclusions:**

Combined CCL2 and CCR2 expression emerges as an independent prognostic factor for non-metastatic ccRCC patients after surgical treatment.

## INTRODUCTION

Renal cell carcinoma (RCC) is the most common type of kidney cancer. During the last two decades, the incidence rate of RCC has increased by 2% annually around the world, afflicting nearly 209,000 people and causing approximately 102,000 deaths every year [1, 2]. The major histologic subtype of RCC is clear-cell RCC, of which more than 10% patients would have fatal recurrence within 5 years after traditional surgical treatment [3]. Currently, several prognostic models based on certain kinds of clinicopathologic features have been established to estimate patients who are at a high risk of relapse after surgery, such as Mayo Clinic stage, size, grade and necrosis (SSIGN) score and University of California Integrated Staging System (UISS) [4]. Although these models have proven to be efficacy in guiding treatment, they still have the potential to be further improved. Previous studies have demonstrated that some molecular biomarkers are capable of distinguishing subtypes of disease and assist in predicting clinical outcomes in various cancer types [5, 6].

Chemokines are a family of small cytokines, or signaling proteins secreted by cells. They together with their receptors mediate inflammatory responses and function as a chemoattractant to guide the migration of leukocytes [7]. However, there is accumulating evidence suggesting that elevated expression of inflammatory chemokines could promote the development of primary tumors as well as metastases [8, 9]. Chemokine (C-Cmotif) ligand 2 (CCL2), also referred to as monocyte chemoattractant protein-1 (MCP1), preferentially binds to the C-C chemokine receptor type 2 (CCR2), which is expressed in various tissues including thymus, lung, liver, kidney, pancreas and ovary [7]. Because of its correlation with the progression of cancer, the CCL2/CCR2 signaling pathway has generated increasing interest in the past few years. It is reported that an elevated expression of CCL2 and CCR2 is observed in a variety of malignancies and is associated with adverse prognosis in patients with breast, nasopharyngeal, colorectal, prostate and pancreatic cancer [10–14]. Blocking CCL2/CCR2 signaling pathway may serve as a novel strategy to help patients with certain kinds of cancers [15]. Actually, RCC is one of the typical malignancies that featured by extensive lo infiltration of inflammatory cells. However, few studies have been carried out to examine the role of CCL2/CCR2 axis in RCC, especially in ccRCC.

Our previous study has demonstrated that CCL2 is an independent adverse prognois factor for post-operative recurrence of ccRCC patients [16]. In the present study, we aim to assess the intratumoral expression of CCL2 as well as CCR2 and determine their prognostic value in ccRCC patients.

## RESULTS

### Intratumoral expression of CCL2/CCR2 and its association with clinicopathological characteristics

**Figure 1:**
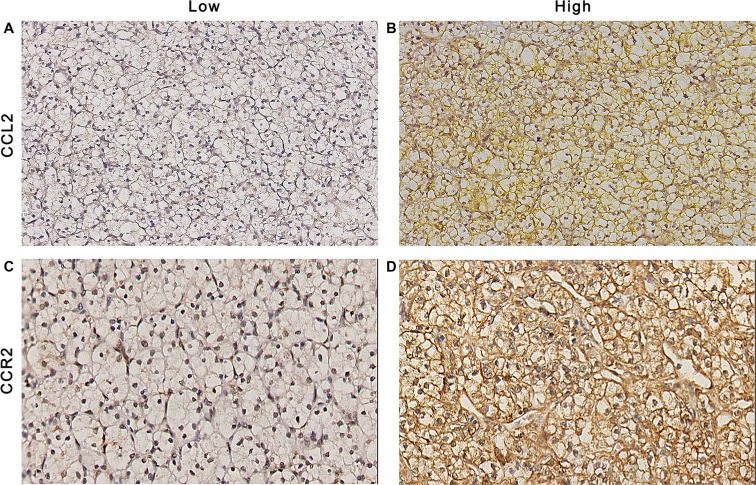
CCL2 and CCR2 expression in ccRCC tissues Representative photograph of CCL2 (**A** and **B**) and CCR2 (**C** and **D**) immunostaining in tissue microarrays. Original magnificent (200×).

To determine whether the expression of CCL2/CCR2 is associated with the development and progression of ccRCC, we analyzed the expression of CCL2 and CCR2 by immunohistochemistry staining in 268 patients at first. CCL2 and CCR2 positive staining were mainly located in the cytoplasm of the tumor cells (Figure 1). According to the cutoff value, 56.0% (150/269) and 63.8% (171/268) of the tumor tissues were scored as high CCL2 and high CCR2 expression, respectively.

Clinicopathologic features for the patients in this research are listed in Table 1. The median age of the patients and median size of the tumor were 56 years, and 4.0 cm, respectively. Tumor necrosis, microvascular invasion and sarcomatoid feature were present in 24.3%, 22.8%, and 6.0% of cases, respectively. Both CCR2 and CCL2 positively correlated with Fuhrman grade (*P* = 0.004 and *P* < 0.001, respectively) and presence of tumor necrosis (*P* < 0.001 and *P* = 0.021, respectively). Tumors from patients in Lebovich HR group tended to express more CCR2 and CCL2 (*P* = 0.005 and *P* = 0.002, respectively).

**Table 1:** Clinical characteristics and correlations with the expression of CCL2 and CCR2

Characteristic	Total patients (*n* = 268)		CCL2			CCR2		
	No.	%	Low (*n* = 150)	High (*n* = 118)	*P* value^a^	Low (*n* = 171)	High (*n* = 97)	*P* value^a^
Age, years Median (IQR)	56 (48–67)		56 (50–67)	55 (45–67)	0.760	56 (49–67)	55 (46–66)	0.401
Gender					0.789			**0.002**
Male	188	29.9	104	84		109	79	
Female	80	70.1	46	34		62	18	
Tumor size, cm Median (IQR)	4.0 (3.0– 6.0)		4.0 (3.0–6.0)	4.0 (3.0–6.0)	0.217	4.0 (3.0–6.0)	4.0 (2.5–6.0)	0.340
PathologicT stage					0.790			0.709
T1a	96	35.8	55	41		64	32	
T1b	74	27.6	41	33		48	26	
T2	34	12.7	21	13		22	12	
T3 + T4	64	23.9	33	31		37	27	
Fuhrman grade					**< 0.001**			**0.004**
1	47	17.5	39	8		39	8	
2	117	43.7	74	43		75	42	
3	67	25.0	29	38		40	27	
4	37	13.8	8	29		17	20	
Tumor necrosis					**0.021**			**< 0.001**
Absent	203	75.7	122	81		143	60	
Present	65	24.3	28	37		28	37	
MVI					0.770			0.010
Absent	207	77.2	117	90		141	66	
Present	61	22.8	33	28		30	31	
Sarcomatoid feature					**< 0.001**			0.553
Absent	252	94.0	148	104		161	91	
Present	16	6.0	2	14		10	6	
UISS score					**0.003**			0.098
LR	100	37.3	69	31		72	28	
IR	153	57.1	72	81		90	63	
HR	15	5.6	9	6		9	6	
Leibovich score					**0.002**			**0.005**
LR	130	48.5	84	46		94	36	
IR	104	38.8	55	49		62	42	
HR	34	12.7	11	23		15	19	

Abbreviations: CCL2 = Chemokine (C-C motif) ligand 2; CCR2 = C-C chemokine receptor type 2; IQR = interquartile range; MVI = microvascular invasion; UISS = University of California Los Angeles Integrated Staging System; LR = low-risk; IR = intermediate-risk; HR = high-risk; No.= number of patients

a A *P* value < 0.05 is considered statistically significant.

### High expression of CCL2 and CCR2 is associated with adverse prognosis

**Figure 2:**
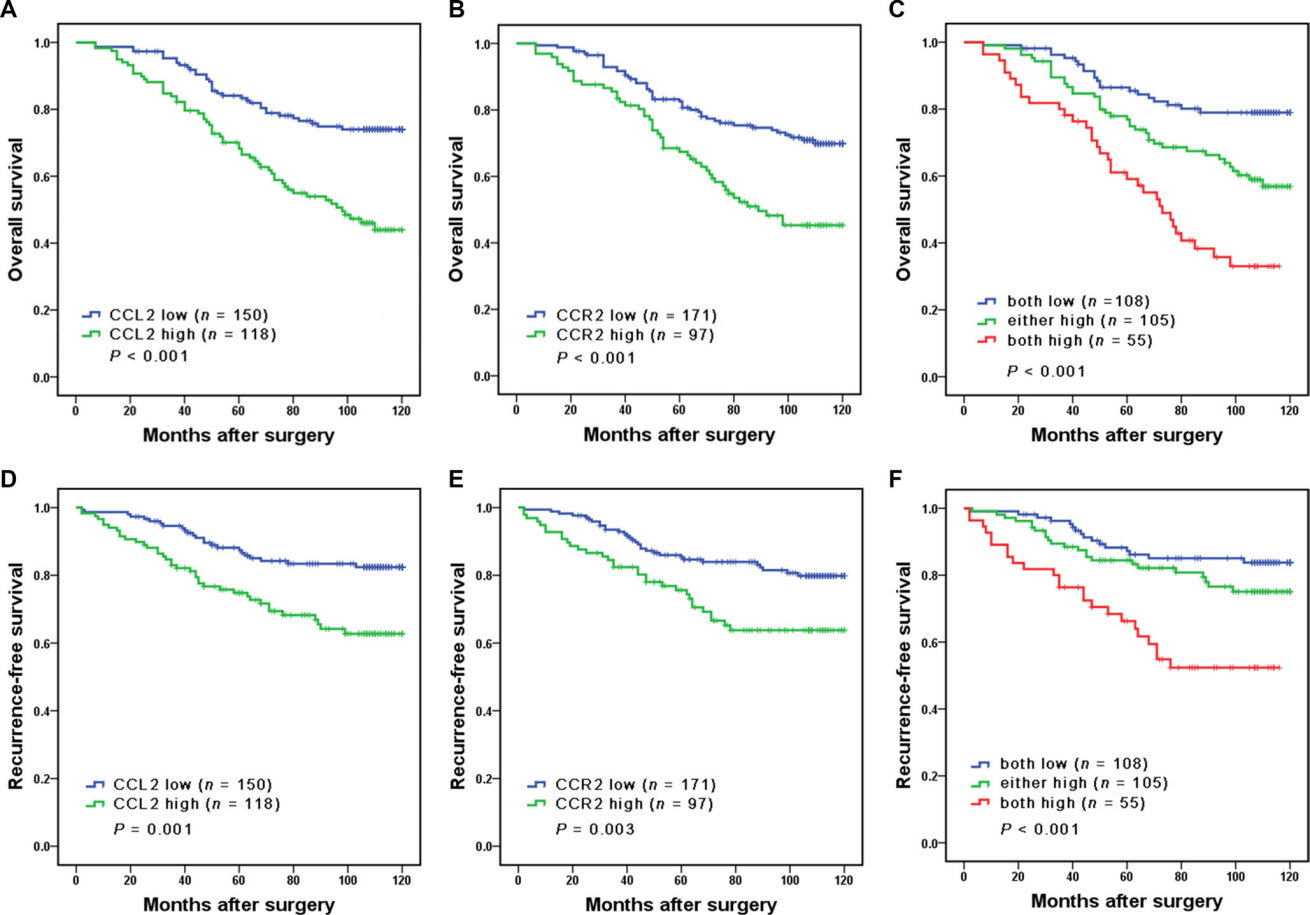
Kaplan Meier analysis of OS (A–C) and RFS (D–F) probabilities based on intratumoral CCL2 and CCR2 expression levels In (C and F), patients were stratified into 3 groups: group I, both low CCL2 and low CCR2 expression; group II, either high CCL2 or high CCR2 expression; group III, both high CCL2 and high CCR2 expression.

Kaplan Meier survival analysis was performed to compare the OS and RFS of the patients, respectively. As single biomarkers, elevated expression of CCL2 and high levels of CCR2 were both remarkably associated with reduced survival (*P* < 0.001 and *P* < 0.001, respectively; Figure 2A, 2B) and increased risk of recurrence (*P* = 0.003 and *P* = 0.001, respectively; Figure 2D, 2E). Furthermore, we found that an improved prognostic stratification of non-metastatic ccRCC patients could be achieved through combined analysis of CCL2 and CCR2. According to their expression levels of CCL2 and CCR2 (named CCL2/CCR2 signature), patients were categorized into three groups: group I, both low CCL2 and low CCR2 expression; group II, either high CCL2 or CCR2 expression; group III, both high CCL2 and CCR2 expression. Significant differences were observed in OS and RFS among the three groups (*P* < 0.001 and *P* < 0.001, respectively; Figure 2C, 2F). The 10-year OS rates for group I, II and III were 80.6%, 61.0% and 38.2%, respectively. The 10-year RFS rates for group I, II and III were 85.2%, 78.1% and 56.4%, respectively.

**Figure 3:**
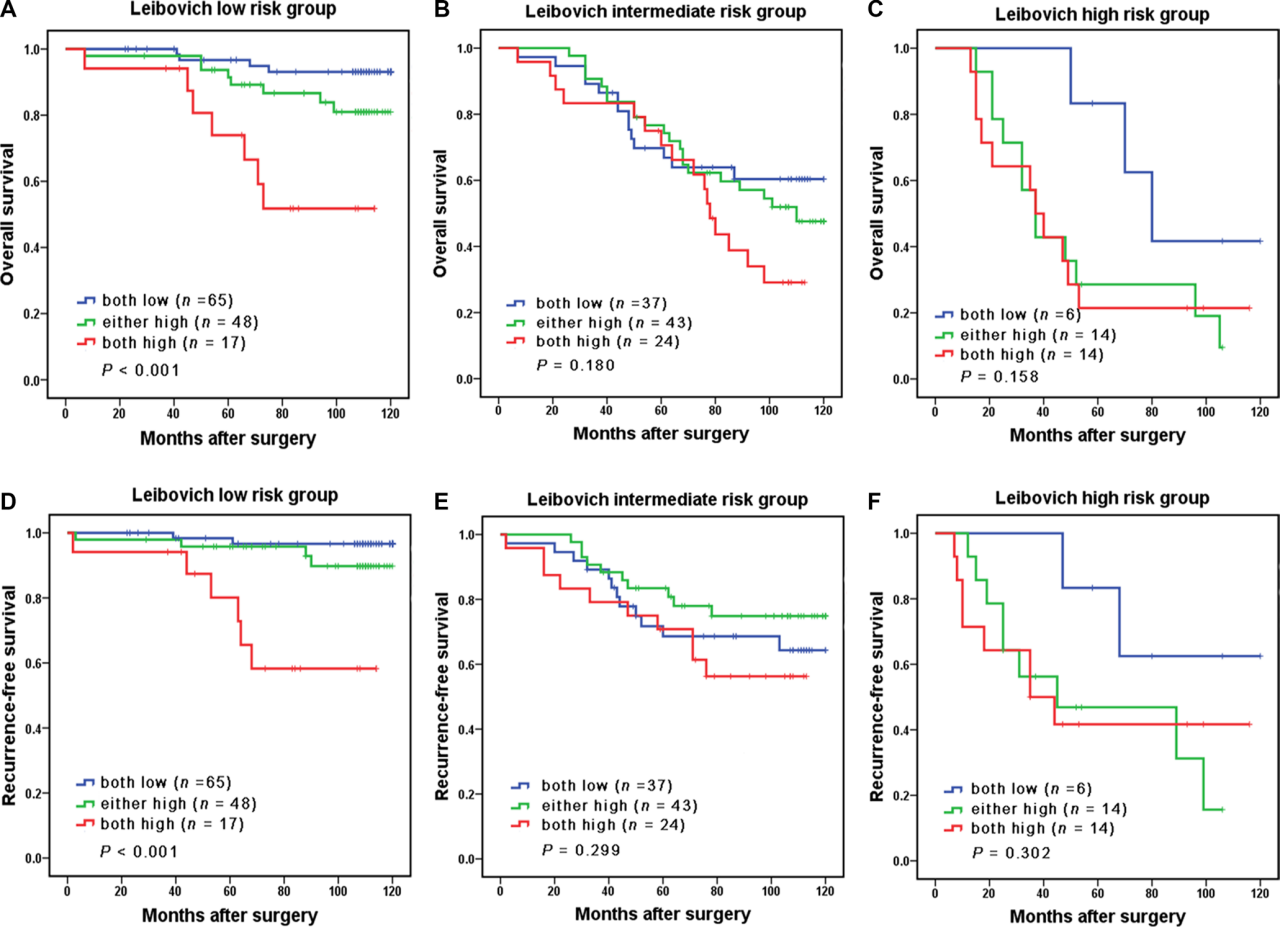
Subgroup analysis to assess prognostic value of CCL2/CCR2 signature in non-metastatic ccRCC patients Kaplan-Meier analysis of OS in patients classified into Leibovich low-risk group (**A**), Leibovich intermediate-risk group (**B**), and Leibovich high-risk group (**C**). Kaplan-Meier analysis of RFS in patients classified into Leibovich low-risk group (**D**), Leibovich intermediate-risk group (**E**), and Leibovich high-risk group (**F**).

We further performed subgroup survival analysis up Leivbovich score model. The Leibovich risk scores of all 268 patients were calculated and classified into three risk groups: low risk (score 0–2; *n* = 130, 48.5%), intermediate risk (score 3–5; *n* = 104, 38.8%), high risk (score ≥ 6; *n* = 34, 12.7%). Significant differences were observed in Leibovich low risk groups (*P* < 0.001 and *P* < 0.001, respectively; Figure 3A, 3D), while no differences were found in Leibovich intermediate and high risk groups (Figure 3B–3F).

### Construction and validation of predictive nomogram for the survival of patients with non-metastatic ccRCC

**Figure 4:**
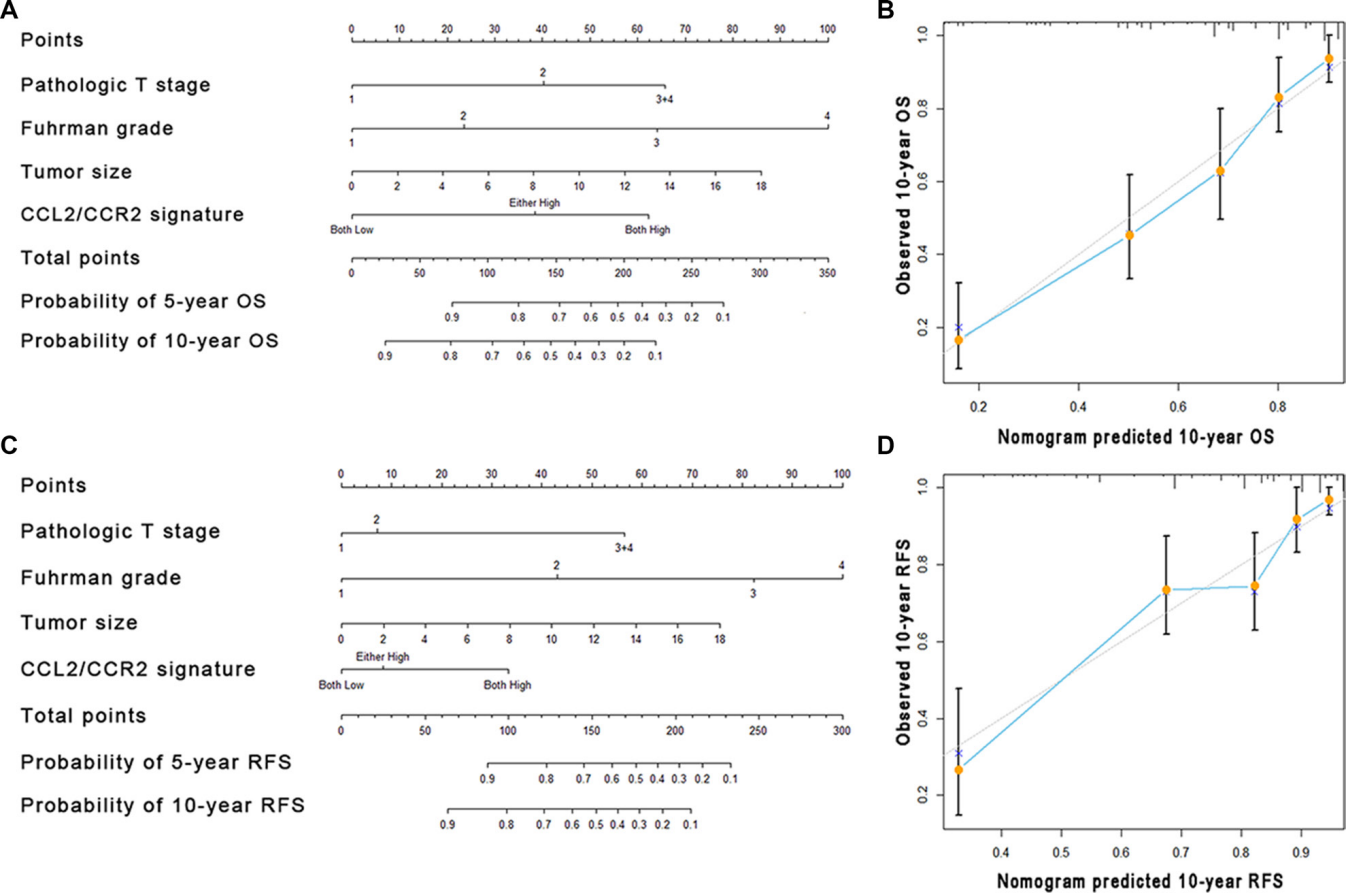
Nomogram and calibration plots for the prediction of outcome in patients with non-metastatic ccRCC Nomogram to predict OS and RFS at 5 and 10 years after nephrectomy (**A** and **C**), the calibration plots for predicting OS and RFS at 10 years (**B** and **D**).

A predictive nomogram, incorporating the independent factors for OS and RFS determined by Cox multivariate analysis, was constructed for the better stratification of patients with different clinicopathological features (Table 2 and Figure 4A, 4C). A higher total point is associated with a worse outcome in the nomogram. The calibration curves were applied to give an internal validation. The 5- and 10-year survival rates of patients predicted by nomogram are in accordance with the ideal model (Figure 4B, 4D). The C-index for the predictive nomogram was 0.770, higher than that for UISS score and Leibovich score (Table 3), indicating that the generated nomogram is an ideal model to predict the survival of non-metastatic ccRCC patients.

**Table 2:** Univariate and multivariate Cox regression analyses for overall survival and recurrence-free survival

variable	Univariate		Multivariate	
	Hazard ratio (95% CI)	*P* value^a^	Hazard ratio (95% CI)	*P* value^a^
Overall survival				
Gender (male vs. female)	0.875 (0.568–1.348)	0.545		
Tumor size (Continuous, cm)	1.175 (1.103–1.251)	**< 0.001**	1.119 (1.045–1.199)	**0.001**
Tumor necrosis (yes vs. no)	1.714 (1.117–2.631)	**0.014**		
Pathologic T stage (3 + 4 vs. 1 + 2)	3.006 (2.001–4.516)	**< 0.001**	2.201 (1.444–3.356)	**< 0.001**
Fuhrman grade (3 + 4 vs. 1 + 2)	3.157 (2.095–4.758)	**< 0.001**	1.783 (1.394–2.230)	**< 0.001**
MVI (present vs. absent)	1.824 (1.189–2.799)	**0.008**		
Sarcomatoid feature (present vs. absent)	3.696 (2.055–6.645)	**< 0.001**		
Combination of CCR2 and CCL2		**< 0.001**		**0.007**
Either high vs. both low	2.183 (1.290–3.695)	**0.004**	1.742 (1.022–2.969)	**0.041**
Both high vs. both low	4.451 (2.576–7.692)	**< 0.001**	2.509 (1.418–4.442)	**0.002**
Recurrence-free survival				
Gender (male vs. female)	0.806 (0.477–1.362)	0.421		
Tumor size (Continuous, cm)	1.169 (1.082–1.263)	**< 0.001**	1.092 (1.002–1.190)	**0.046**
Tumor necrosis (yes vs. no)	2.016 (1.206–3.368)	**0.007**		
Pathologic T stage (3 + 4 vs. 1 + 2)	4.217 (2.569–6.920)	**< 0.001**	3.098 (1.855–5.173)	**< 0.001**
Fuhrman grade (3 + 4 vs. 1 + 2)	3.731 (2.220–6.271)	**< 0.001**	1.899 (1.421–2.537)	**< 0.001**
MVI (present vs. absent)	2.012 (1.198–3.379)	**0.008**		
Sarcomatoid feature (present vs. absent)	4.196 (2.131–8.264)	**< 0.001**		
Combination of CCR2 and CCL2		**< 0.001**		**0.043**
Either high vs. both low	1.573 (0.831–2.979)	0.164	1.231(0.645–2.349)	0.529
Both high vs. both low	3.831 (2.030–7.230)	**< 0.001**	2.057(1.061–3.989)	**0.033**

Abbreviations: CCL2 = Chemokine (C-C motif); CCR2 = C-C chemokine receptor type 2; MVI = microvascular invasion; CI = confidence interval; HR = hazard ratio.

a A *P* value < 0.05 is considered statistically significant.

**Table 3:** Comparison of the prognostic accuracies of models for OS and RFS

Models	Overall survival		Recurrence-free survival	
	C-index	AIC	C-index	AIC
UISS score	0.658	976.941	0.676	639.015
Leibovich score	0.724	957.268	0.742	629.211
CCR2/CCL2	0.645	990.286	0.638	661.063
UISS+ CCR2/CCL2	0.714	957.684	0.724	629.723
Leibovich score+ CCR2/CCL2	0.750	944.651	0.762	624.162
Predictive nomogram	0.770	937.632	0.785	615.755

Abbreviations: C-index = Harrell's concordance index, AIC = Akaike information criterion, UISS = University of California Los Angeles Integrated Staging System,

An elevated C-index or a decreased AIC score means a better prognosis.

### Extension of prognostic models with CCL2/CCR2 expression

Apart from TNM stage, the UISS score and Leibovich score are widely used to estimate prognosis after surgical treatment for RCC patients. We combined CCL2/CCR2 signature with the mentioned models to determine whether the accuracy of the predictive models could be improved. For OS, the C-indices of the UISS score and Leibovich score were originally 0.658, 0.724 respectively, and increased to 0.714, 0.750 (*P* = 0.002 and *P* = 0.010, respectively). For RFS, the C-indices of the two models were originally 0.676, 0.742 respectively, and then risen to 0.724, 0.762 (*P* = 0.007 and *P* = 0.280, respectively) (Table 3).

## DISCUSSION

In this study, we have demonstrated that the combination of CCL2 and CCR2 is an independent risk factor for patients with non-metastatic ccRCC. Elevated expression of intratumoral CCL2 and CCR2 is significantly associated with shortened survival time and increased risk of recurrence. Of particular interest, subgroup analysis revealed that CCL2/CCR2 signature could further stratify patients in low risk groups defined by Leibovich score. In addition, the incorporation of CCL2/CCR2 signature into UISS score and Leibovich score would improve the predictive accuracy of these models. In order to further strengthen the prognostic value of CCL2/CCR2 signature for clinical outcome, we developed a postoperative nomogram to predict long-term overall survival and recurrence-free survival of non-metastatic ccRCC patients based on 10-year follow-up.

Previous studies have demonstrated that inflammatory environment, which primarily consists of inflammatory cells and inflammatory cytokines, is involved in cancer progression and metastasis [17–19]. Among the inflammatory cells, macrophages are especially abundant and could be observed at the entire period of tumor progression [20]. It has been reported that CCL2 and its primary receptor CCR2 have the ability to recruit monocyte from peripheral blood to tumor site regulate the mobilization of macrophages [21, 22]. In addition, CCL2/CCR2 signaling could educate tumor-associated macrophages (TAMs) to enhance the production of several kinds of immunosuppressive cytokines and chemokines, which in turn facilitate the progression of tumors and exert an unfavorable effect on cancer patients [23]. In murine models, it has been verified that blocking CCL2/CCR2 signal pathway could restraint the infiltration of macrophages and postpone cancer metastasis [22]. Thus, it is not surprising to observe that an elevated expression of CCL2/CCR2 is present in several cancer types and is associated with adverse clinical outcomes [12–14, 24].

Apart from recruiting and educating TAMs, CCL2/CCR2 has been reported to be responsible for the accumulation of myeloid-derived suppressor cells (MDSCs) in tumor sites [15]. MDSCs, which mainly consists of immature myeloid progenitors for monocytes, dendritic cells and neutrophils, could promote tumor growth not only through suppressing immune responses, but also through establishing supportive microenvironment such as angiogenesis for neoplastic progression [25]. Another study suggested that CCL2/CCR2 axis could promote cancer metastasis by up-regulation of MMP2/9 through ERK1/2 signaling pathway [11]. However, these studies were performed in other cancer models, the mechanism that CCL2/CCR2 signaling contributes to the unfavorable outcomes of ccRCC patients remains to be fully understood.

As given the prognostic value CCL2/CCR2 signature in non-metastatic ccRCC, the molecules involved in the pathway might have the potential to become the novel therapeutic targets for precise treatment of ccRCC as well. It has been reported that targeting CCL2/CCR2 signaling axis remarkably reduced the motility and survival of breast cancer cells [10]. And In mice, CCL2/CCL2 axis blockade lessened inflammatory monocytes and macrophages from the primary tumor site and pre-metastatic liver, leading to enhanced antitumor immunity, regressed tumor growth, and delayed metastasis [24]. Thus, the blockade of the CCL2/CCR2 signaling pathway to treat renal cancer is a promising direction and deserves further investigation.

There are several limitations of this study that warrant further discussion. First, because all of the patients enrolled in our research are from Asia, the result of this study needs to be validated in other populations and larger cohorts. Second, the TMA technique only displays a small piece of the original tumor tissue, the information contained may not be typical and the expression of CCL2 and CCR2 is evaluated subjectively. Third, the predictive value of CCL2 and CCR2 expression is simply verified in non-metastatic ccRCC owing to limited cases with metastasis. Further assessment in metastatic RCC needs to be performed in the future.

In conclusion, we have identified the increased expression of CCL2/CCR2 in non-metastatic ccRCC as an independent unfavorable prognostic factor, which could be integrated with pathologic T stage, Fuhrman grade and the size of the tumor to generate a nomogram to give a better risk stratification for patients with different prognosis. Inhibiting CCL2/CCR2 signaling pathway might be a promising novel therapy for ccRCC patients.

## MATERIALS AND METHODS

### Patients

We retrospectively enrolled 268 patients with non-metastatic ccRCC who underwent surgical treatment at Zhongshan Hospital of Fudan University (Shanghai, China) from 2001 to 2004. None of the patients suffered from other types of renal cancer or had a history of previous anti-cancer therapy. After surgery, patients accepted physical examination, laboratory diagnosis, chest imaging, abdominal CT scans or ultrasound twice a year for the first two years and annually thereafter. Patients were followed up to 10 years after surgery. The followed-up period ranged from 12 to 120 months and the median period was 89 months. OS and RFS were measured from the date of surgery to the date of death and recurrence, respectively or to the date of last follow-up. For each patient, we gathered the following clinicopathologic information: age, gender, size of the tumor, TNM stage, Fuhrman grade, presence of tumor necrosis, microvascular invasion, and sarcomatoid feature. On the basis of radiographic reports and postoperative pathologic data, all of the patents were staged and then reassigned according to the 2010 American Joint Committee on Cancer TNM classification [26]. The UISS predictive models and the Leibovich prognostic score were used to categorize patients into 3 different risk groups. This study was approved by the research medical ethics committee of Zhongshan hospital and was carried out in strict accordance with the approved guidelines.

### Tissue microarray and immunohistochemistry

The construction of tissue microarray and the immunohistochemical procedure were performed as previously described [27]. Primary Anti-CCL2 antibody (diluted 1:200; ab9669, Abcam) and Anti-CCR2 antibody (diluted 1:200; ab32144, Abcam) were used for immunohistochemical staining. The immunostaining intensity was evaluated independently by two pathologists without any knowledge of the patients’ outcomes and clinicopathological features. A semi-quantitative score, which ranges from 0 to 300, was used to describe the intensity of staining. It was calculated by multiplying the staining intensities (0: negative; 1: weak staining; 2: moderate staining; 3: strong staining) by the area distributions (0–100%).

### Statistical analysis

The minimum *P* value approach calculated by X-tile software (Yale University, New Haven, CT) was applied to obtain the optimal cutoff that separated each cytokine into low and high expression related to the patients’ OS. Statistical analysis was performed using SPSS 21.0 (IBM Corporation, Armonk, NY, USA), R software version 3.0.2 and the ‘rms’ package (R Foundation for Statistical Computing, Vienna, Austria). Categorical variables were analyzed by Pearson χ2-test while continuous variables were compared by *t* test. Kaplan-Meier method and the log-rank test were used to establish the survival curves. The Cox proportional hazards regression model was applied to perform univariate and multivariate analyses. The accuracy of multivariate models was measured by the Harrell's Concordance index (C-index), which is calculated by Stata 12.0 (Stata CorpLP, College Station, TX). All statistical tests were two-sided and were performed at a significance level of *P* < 0.05.

## References

[1] Gupta K, Miller JD, Li JZ, Russell MW, Charbonneau C (2008). Epidemiologic and socioeconomic burden of metastatic renal cell carcinoma (mRCC): a literature review. Cancer Treat Rev.

[2] Ljungberg B, Bensalah K, Canfield S, Dabestani S, Hofmann F, Hora M, Kuczyk MA, Lam T, Marconi L, Merseburger AS, Mulders P, Powles T, Staehler M (2015). EAU guidelines on renal cell carcinoma: 2014 update. Eur Urol.

[3] Ljungberg B, Campbell SC, Choi HY, Jacqmin D, Lee JE, Weikert S, Kiemeney LA (2011). The epidemiology of renal cell carcinoma. Eur Urol.

[4] Sun M, Shariat SF, Cheng C, Ficarra V, Murai M, Oudard S, Pantuck AJ, Zigeuner R, Karakiewicz PI (2011). Prognostic factors and predictive models in renal cell carcinoma: a contemporary review. Eur Urol.

[5] Eichelberg C, Junker K, Ljungberg B, Moch H (2009). Diagnostic and prognostic molecular markers for renal cell carcinoma: a critical appraisal of the current state of research and clinical applicability. Eur Urol.

[6] Parker AS, Leibovich BC, Lohse CM, Sheinin Y, Kuntz SM, Eckel-Passow JE, Blute ML, Kwon ED (2009). Development and evaluation of BioScore: a biomarker panel to enhance prognostic algorithms for clear cell renal cell carcinoma. Cancer.

[7] Lim S, Yuzhalin A, Gordon-Weeks A, Muschel R (2016). Targeting the CCL2-CCR2 signaling axis in cancer metastasis. Oncotarget.

[8] Balkwill F (2004). Cancer and the chemokine network. Nat Rev Cancer.

[9] Zlotnik A (2006). Chemokines and cancer. Int J Cancer.

[10] Fang WB, Jokar I, Zou A, Lambert D, Dendukuri P, Cheng N (2012). CCL2/CCR2 chemokine signaling coordinates survival and motility of breast cancer cells through Smad3 protein- and p42/44 mitogen-activated protein kinase (MAPK)-dependent mechanisms. J Biol Chem.

[11] Yang J, Lv X, Chen J, Xie C, Xia W, Jiang C, Zeng T, Ye Y, Ke L, Yu Y (2016). CCL2-CCR2 axis promotes metastasis of nasopharyngeal carcinoma by activating ERK1/2-MMP2/9 pathway. Oncotarget.

[12] Zhao L, Lim SY, Gordon-Weeks AN, Tapmeier TT, Im JH, Cao Y, Beech J, Allen D, Smart S, Muschel RJ (2013). Recruitment of a myeloid cell subset (CD11b/Gr1mid) via CCL2/CCR2 promotes the development of colorectal cancer liver metastasis. Hepatology.

[13] Lu Y, Chen Q, Corey E, Xie W, Fan J, Mizokami A, Zhang J (2009). Activation of MCP-1/CCR2 axis promotes prostate cancer growth in bone. Clin Exp Metastas.

[14] Monti P, Leone BE, Marchesi F, Balzano G, Zerbi A, Scaltrini F, Pasquali C, Calori G, Pessi F, Sperti C (2003). The CC chemokine MCP-1/CCL2 in pancreatic cancer progression regulation of expression and potential mechanisms of antimalignant activity. Cancer Res.

[15] Huang B, Lei Z, Zhao J, Gong W, Liu J, Chen Z, Liu Y, Li D, Yuan Y, Zhang GM, Feng ZH (2007). CCL2/CCR2 pathway mediates recruitment of myeloid suppressor cells to cancers. Cancer Lett.

[16] Yang Y, Zhai C, Chang Y, Zhou L, Shi T, Tan C, Xu L, Xu J (2016). High expression of chemokine CCL2 is associated with recurrence after surgery in clear-cell renal cell carcinoma. Urol Oncol.

[17] Coussens LM, Werb Z (2002). Inflammation and cancer. Nature.

[18] Grivennikov SI, Greten FR, Karin M (2010). Immunity, inflammation, and cancer. Cell.

[19] Kim S, Takahashi H, Lin W-W, Descargues P, Grivennikov S, Kim Y, Luo J-L, Karin M (2009). Carcinoma-produced factors activate myeloid cells through TLR2 to stimulate metastasis. Nature.

[20] Noy R, Pollard JW (2014). Tumor-associated macrophages: from mechanisms to therapy. Immunity.

[21] Pollard JW (2004). Tumour-educated macrophages promote tumour progression and metastasis. Nat Rev Cancer.

[22] Qian B-Z, Li J, Zhang H, Kitamura T, Zhang J, Campion LR, Kaiser EA, Snyder LA, Pollard JW (2011). CCL2 recruits inflammatory monocytes to facilitate breast-tumour metastasis. Nature.

[23] Li X, Yao W, Yuan Y, Chen P, Li B, Li J, Chu R, Song H, Xie D, Jiang X (2015). Targeting of tumour-infiltrating macrophages via CCL2/CCR2 signalling as a therapeutic strategy against hepatocellular carcinoma. Gut.

[24] Sanford DE, Belt BA, Panni RZ, Mayer A, Deshpande AD, Carpenter D, Mitchem JB, Plambeck-Suess SM, Worley LA, Goetz BD (2013). Inflammatory monocyte mobilization decreases patient survival in pancreatic cancer: a role for targeting the CCL2/CCR2 axis. Clin Cancer Res.

[25] Murdoch C, Muthana M, Coffelt SB, Lewis CE (2008). The role of myeloid cells in the promotion of tumour angiogenesis. Nat Rev Cancer.

[26] Edge SB, Compton CC (2010). The American Joint Committee on Cancer: the 7th edition of the AJCC cancer staging manual and the future of TNM. Ann Surg Oncol.

[27] Zhu XD, Zhang JB, Zhuang PY, Zhu HG, Zhang W, Xiong YQ, Wu WZ, Wang L, Tang ZY, Sun HC (2008). High expression of macrophage colony-stimulating factor in peritumoral liver tissue is associated with poor survival after curative resection of hepatocellular carcinoma. J Clin Oncol.

